# Development of a Tonometric Sensor with a Decoupled Circular Array for Precisely Measuring Radial Artery Pulse

**DOI:** 10.3390/s16060768

**Published:** 2016-05-26

**Authors:** Min-Ho Jun, Young-Min Kim, Jang-Han Bae, Chang Jin Jung, Jung-Hee Cho, Young Ju Jeon

**Affiliations:** KM Fundamental Research Division, Korea Institute of Oriental Medicine (KIOM), Daejeon 305-811, Korea; mino@kiom.re.kr (M.-H.J.); irobo77@kiom.re.kr (Y.-M.K.); fcbest11@kiom.re.kr (J.-H.B.); traviswas@kiom.re.kr (C.J.J.); xtin@kiom.re.kr (J.-H.C.)

**Keywords:** pressure sensor, radial artery pulse, piezoresistive sensor, tonometric sensor, face-down bonding (FDB), decoupled circular array

## Abstract

The radial artery pulse is one of the major diagnostic indices used clinically in both Eastern and Western medicine. One of the prominent methods for measuring the radial artery pulse is the piezoresistive sensor array. Independence among channels and an appropriate sensor arrangement are important for effectively assessing the spatial-temporal information of the pulse. This study developed a circular-type seven-channel piezoresistive sensor array using face-down bonding (FDB) as one of the sensor combination methods. The three-layered housing structure that included independent pressure sensor units using the FDB method not only enabled elimination of the crosstalk among channels, but also allowed various array patterns to be created for effective pulse measurement. The sensors were arranged in a circular-type arrangement such that they could estimate the direction of the radial artery and precisely measure the pulse wave. The performance of the fabricated sensor array was validated by evaluating the sensor sensitivity per channel, and the possibility of estimating the blood vessel direction was demonstrated through a radial artery pulse simulator. We expect the proposed sensor to allow accurate extraction of the pulse indices for pulse diagnosis.

## 1. Introduction

The radial artery pulse has long been one of the major diagnostic methods in both Eastern and Western medicine [1,2,3]. Two thousand years ago, the Greek medical scientist Galen classified pulse waves and used them for clinical diagnoses, and his diagnosis theory remained in use until the medieval era. Since the invention of the sphygmogram in 1863, it has been possible to measure pulse waves to utilize various variables, such as blood pressure (BP) and augmentation index (AIx), and it is widely used in clinics [4,5,6,7].

Currently, there are many requests to develop objective and scientific equipment for precisely measuring pulse waves on radial arteries [8,9,10]. The shape characteristics of the pulse reflect the condition of the cardiovascular system, such as the elasticity and viscosity of the arterial wall, compliance, and the resistance of the blood flow. In Eastern medicine, traditional doctors analyze not only the temporal, but also the spatial, characteristics of the pulse wave on the left and right radial arteries to diagnose diseases and discern the effectiveness of treatments [11,12]. These doctors have been using their fingers to measure the detailed shape of pulse waves, although the diagnosis result can be highly dependent on the senses of the doctors. The detailed shape characteristics of the pulse wave are difficult to obtain using general-purpose measurement systems. Measuring the spatial-temporal characteristics of the pulse wave with various conditions of applying pressure to the radial artery provides more useful information for diagnosis than conventional measurement methods. Therefore, an advanced measurement system needs to be developed for measuring the detailed properties of the cardiovascular system and for accurate diagnoses in Eastern medical clinics.

Various types of sensing elements for effective pulse measurements, such as piezoelectric, piezoresistive, capacitive, and hall sensors, have been steadily developed. These elements have their own advantages in terms of their measurement principle; however, they have problems that must be overcome for precisely measuring pulse waves on radial arteries. The pulse sensor that uses piezoelectric elements is limited to obtaining static information, which means the applied pressure on the radial artery. The acquisition of static information is important for precisely measuring pulse waves because the shape of the pulse changes according to the applied pressure [13]. Hall sensors measure changes in the magnetic field generated by the movement of permanent magnets due to pulsation of the radial artery. Although hall sensors have the advantage of measuring dynamic information of pulse waves in a small area, these sensors are not suitable for measuring static data [14]. Pulse-taking platforms that use a capacitive tactile sensor array (Pressure Profile System Company, Los Angeles, CA, USA) can measure pulse length and width using 3-D pulse wave data obtained through a 3 × 4 array of capacitive sensors. However, the signal interference among channels has yet to be solved, and the low sensitivity (over 0.5 mmHg in the full sensing range of 300 mmHg) makes observing subtle changes in the pressure pulse waveform difficult [15].

The piezoresistive sensor is the prominent method for precisely measuring radial artery pulses because it can acquire both static and dynamic information of pulse waves with high sensitivity. The piezoresistive sensor requires a temperature compensation for signals changed by the heat conducted from the skin. A study by Yoo *et al.* [16] developed a pulse sensor that includes a temperature sensor to compensate the output signals according to temperature changes. The crosstalk among channels of a sensor array is an important issue for measuring both the temporal and spatial information of the pulse wave for pulse diagnosis. Various types of sensor arrays, including piezoresistive sensors, contain crosstalk, and it decreases the measurement accuracy of each channel. Therefore, sensors need to be designed that minimize crosstalk among channels. 

In this study, we developed an advanced sensor for precisely measuring pulse waves on radial arteries and a method for improving its performance. A circular-type pulse sensor array with seven piezoresistive sensors was developed using a fabrication method with face-down bonding (FDB). Crosstalk among channels was eliminated by the sensor design, which can transmit pressure for each channel independently. By using a stick pad as an insulator, the sensor can only obtain the signals regardless of temperature changes. In addition, a method for estimating the direction of the radial artery was proposed to provide the proper direction for the sensor pressure and to improve the measurement accuracy. The sensitivity and coefficient of variance (CV) of the proposed sensor were assessed experimentally, and the performance of the method for estimating artery direction was evaluated using a radial artery pulse simulator. The estimation of artery direction facilitates more accurate analysis for the spatial information of the pulse, such as depth, width, and force.

## 2. Design and Experimental Section

### 2.1. FDB-Type Pressure Sensor for Radial Pulse (PSRP)

#### 2.1.1. The Structure and Assembly of PSRP

**Figure 1:**
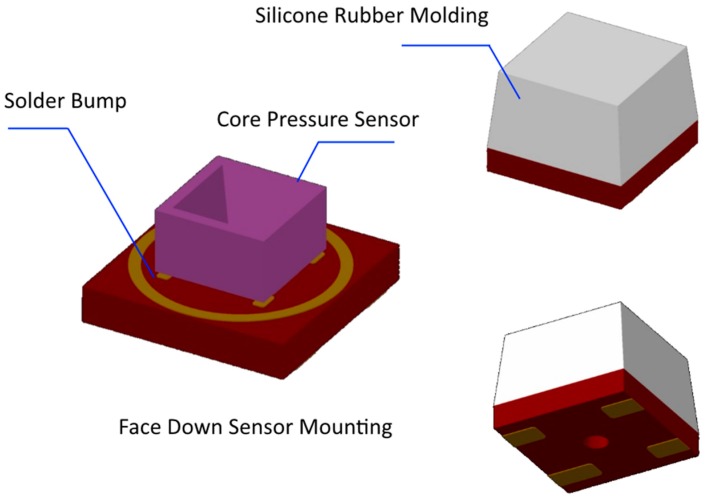
Independent pressure sensor unit (IPU) size: 2.5 mm × 2.5 mm × 2.7 mm.

A gauge-type silicon pressure sensor (US9173, Unisense, Hsinchu, Taiwan) was used to measure the pulse pressure of the radial artery. This sensor was integrated with the PCB by FDB, and it was covered with a silicone rubber mold. Independent pressure sensor units (IPUs) were composed of PCB, core pressure sensor, and silicone rubber mold, as shown in Figure 1. The core sensor was connected to the PCB by a solder bump. This process increases the durability of the sensor, and it improves the degree of integration of the sensor due to the wireless device.

**Figure 2:**
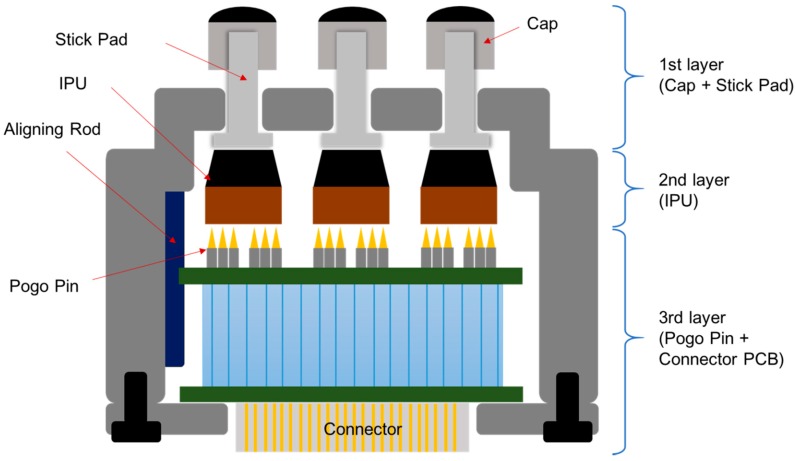
PSRP structure.

**Figure 3:**
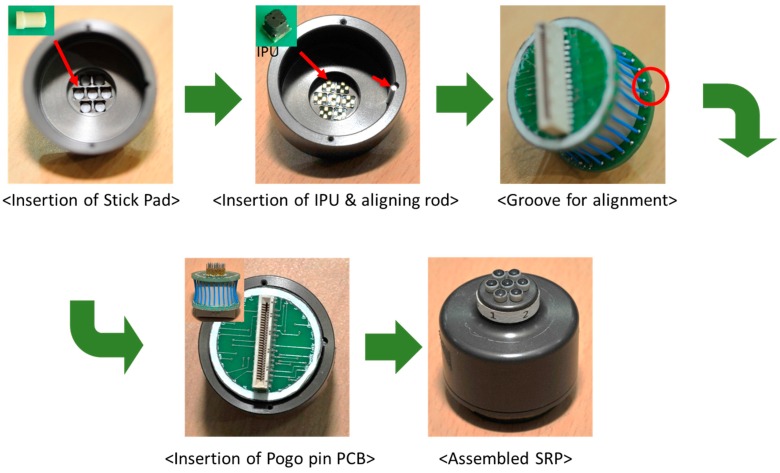
PSRP assembly process.

Figure 2 shows the structure of the PSRP. The PSRP has three layers: the first layer consists of a stick pad for delivering the pulse of the radial artery to the IPU, as well as the cap on the stick pad. Each stick pad is mounted on an IPU and protrudes externally through the hole in the outer case of the PSRP. This structure enables the crosstalk between IPUs to be eliminated because the radial artery pulse is transmitted to the IPU individually. The cap was used to broaden the contact surface of the skin. This sensor has the structural advantage of minimizing the effect of temperature changes because the stick pad insulates against heat from the skin. The second layer is the IPU, and the third layer consists of the connector PCB and pogo pins for connecting the IPU and the PCB. There is a groove for alignment to obtain a precise connection between the pogo pins and IPUs. Figure 3 shows the assembly process for this PSRP. This sensor is cost effective because a malfunctioning IPU is simply replaced with a new one rather than replacing the entire PSRP.

**Figure 4:**
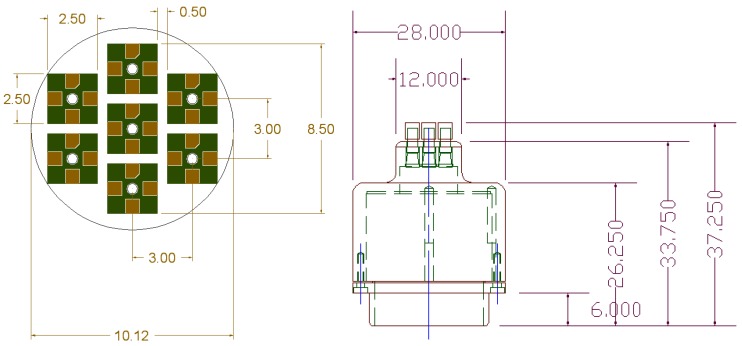
PSRP Dimensions (unit: mm).

Figure 4 shows the dimensions of the developed PSRP. IPUs are placed at 0.5 mm gaps, and the diameter of the first layer, which consists of seven IPUs, stick pads, and caps, is 10.12 mm. The IPUs can precisely measure parallel and vertical pulse information of the radial artery because seven IPUs were placed in a circular arrangement, as shown in Figure 4. The experimental results also demonstrated that the developed PSRP was a suitable structure for assessing the spatial characteristics of the pulse wave.

#### 2.1.2. PSRP Evaluation

**Figure 5:**
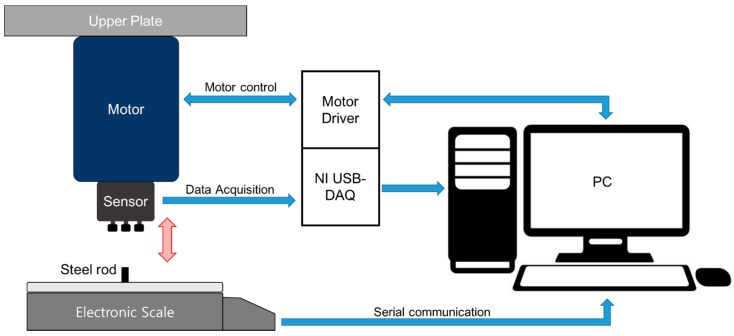
Schematic diagram of the sensor assessment system.

The PSRP evaluation system can apply a force of seven steps on each channel to evaluate the characteristics of the PSRP. The system consists of three parts: an actuator that applies force on each channel using a motor, an electronic scale (CUX4200H, CAS, Seongnam-si, Korea) that can measure the mass applied to the channel, and a controller that controls motor movements according to the input from the electronic scale through serial communication. Figure 5 presents a diagram of the sensor assessment system.

The evaluation system automatically applies force (*i.e.*, 0 gf, 5 gf, 10 gf, 15 gf, 20 gf, 25 gf, and 30 gf) to each channel until the target force is reached. The sensor signals are stored in a PC via a DAQ (NI-USB6218, National Instruments, Austin, TX, USA). The seven-step force was sequentially applied, and the sensor signals and output from the electronic scale were saved. The sensor assessment system repeated this process three times. Considering the contact area (3.61 mm^2^) of the cap connected with each channel, a target force of 10 gf is equivalent to a pressure of 203.76 mmHg. The sensitivity was evaluated using each channel output signal. From the three repeated measurements, the repeatability was calculated employing the coefficient of variance (CV).

### 2.2. Method for Estimating Blood Vessel Direction

**Figure 6:**
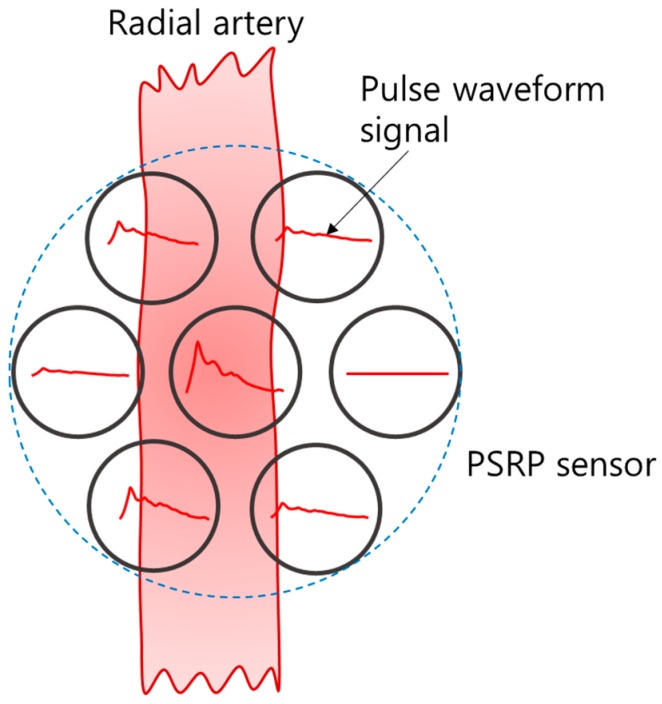
Schematic diagram for estimating blood vessel direction using PSRP.

The developed PSRP is less influenced by skin temperature and interference among channels, and it is suitable for estimating the blood vessel direction due to the circular-shaped arrangement. Figure 6 presents a schematic diagram of the method for estimating the blood vessel direction using PSRP on the radial artery. When a channel is at the center artery, it has a high-amplitude signal, and the signal becomes smaller as a channel is far from the center. To develop the method for estimating the direction of the radial artery, an experiment for finding the blood vessel direction was performed using a pulse wave simulator (Victor Pulse, Tellyes Scientific Inc., Tianjin, China).

**Figure 7:**
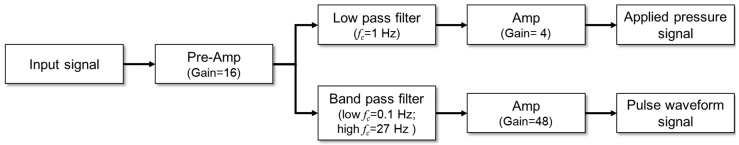
Flow chart of data acquisition of applied pressure and pulse waveform.

The output signals from the experiment were collected using the following process. The PSRP, which has seven channels arrayed in a circular type arrangement, is rotated at a certain angle. The output signals of the PSRP were obtained as the channels that are applied with a predetermined force. The direction of the blood vessel was estimated through the analysis of the output signals. The signals obtained through the channels were pre-amplified and divided into two parts: applied pressure signals and pulse waveform signals, as shown in Figure 7. The applied pressure signals and the pulse waveform signals can be obtained by passing the input signals through a low pass filter and band pass filter, respectively.

**Figure 8:**
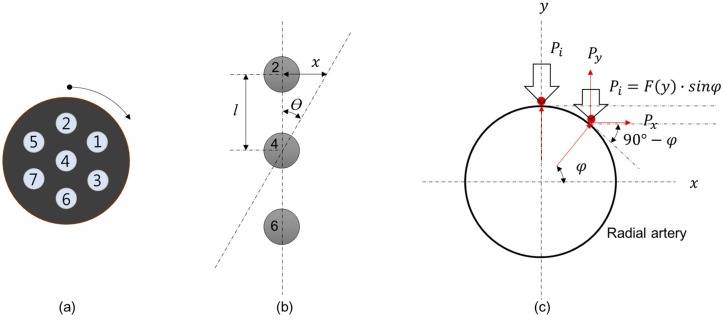
PSRP model and artery model pushed by channels: (**a**) PSRP of the decoupled circular type with rotation (*Ɵ*), (**b**) location change of channels rotated around channel four, and (**c**) the load on arterial vessel wall pushed by channels.

Figure 8 shows a geometrical model of the PSRP and blood vessel for estimating blood vessel direction through sensor rotation. Figure 8a depicts the entire circular-type sensor and the channel number of each channel. Figure 8b presents a geometrical model for the moving distance of the channels from the *x*-axis when the channels that are in a straight line (*i.e.*, two, four, and six) are rotated around channel four clockwise (CW). Figure 8c depicts the direction and amplitude of the various forces applied to the radial artery (assumed to be a circular shape). The force is reduced by the geometric shape and force direction as the channels are moved from the *x*-axis by the rotation of the PSRP.

In other words, when the PSRP rotates around its center point (channel four), the channels above and below channel four move from the *x*-axis as much as the rotation angle. When the channel applies pressure to the lowest vessel wall at the distance moved from the center of the vessel, the force that the channel receives is decreased according to Hooke’s Law.

Assuming that the blood vessel is circular (with the equation of a circle: $x^{2}+y^{2}=r^{2}$, where *r* is the radius of a circle) and that the output values of the channel are proportional to the height difference from the center of the blood vessel (by Hooke’s Law, *F* = k*x*, where k is a constant), the normalized expression of the force ($P_{y}$) obtained at the channel is in the following form:

(1) $$P_{y}\;=\left\{F\left(y\right)\cdot sin\varphi -F\left(y\right)\cdot cos^{2}\varphi \right\}\cdot sin\varphi$$

Here, F(y) is the correlation between the height of the channel and the applied force, $y_{min}$ is the height at which F(y) becomes 0, and $y_{max}$ is the height at which the channel is located at the center of the blood vessel. F(y) can be expressed as follows:

(2) $$F\left(y\right)=k\cdot (y_{max}-y_{min})$$

where k is the spring constant. F(y) is 1 when the channel is at the center of the blood vessel, and F(y) is 0 when the channel is located at $y_{min}$. The location of the channel that applies pressure to the blood vessel’s wall (x, y, and angle (φ)) is obtained by the following, using the equation of a circle and trigonometric functions:

(3) $$x=l\cdot sin\theta ,\;y=\sqrt{r^{2}-{\left(l\cdot sin\theta \right)}^{2}},\;\varphi =tan^{-1}\frac{y}{x}$$

The obtained force F(y) and angle (φ) are substituted into Equation (1) to obtain the pressure applied by the channel according to location. Finally, the equation for normalized pressure ($P_{y}$) according to PSRP rotation (θ) can be expressed as:

(4) Py={k·(r2−(l·sinθ)2−ymin)·sin(tan−1r2−(l·sinθ)2l·sinθ)−kPy={k·(r2−·(r2−(l·sinθ)2−ymin)·cos2(tan−1r2−(l·sinθ)2l·sinθ)}Py={k·(r2−·sin(tan−1r2−(l·sinθ)2l·sinθ)

**Figure 9:**
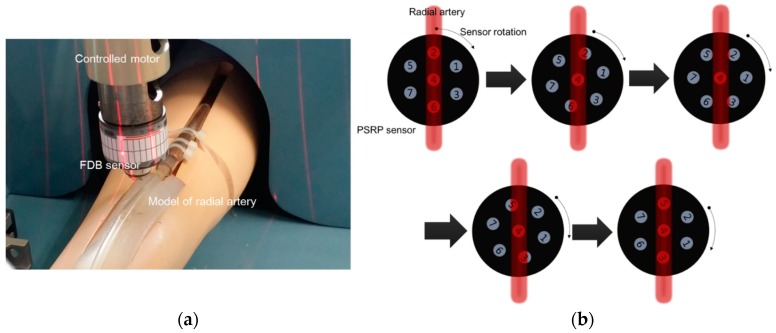
(**a**) Experimental set-up and (**b**) experimental sequence for estimating blood vessel direction.

An experimental set-up was established to confirm the possibility of estimating the blood vessel’s direction, as shown in Figure 9a. First, the produced PSRP was located on top of the pulse wave simulator of the radial artery, which generates pulse waves, and signals were obtained by repeatedly applying pressure and rotation. A DC motor was used to control the desired pressure applied by the output signal of channel 4 in the PSRP, and the rotation angle was manually controlled using a scaled ruler on the outer case of the PSRP and a compact structured light laser (3D PRO Laser Mini^™^, ProPhotonix, Salem, NH, USA).

**Figure 10:**
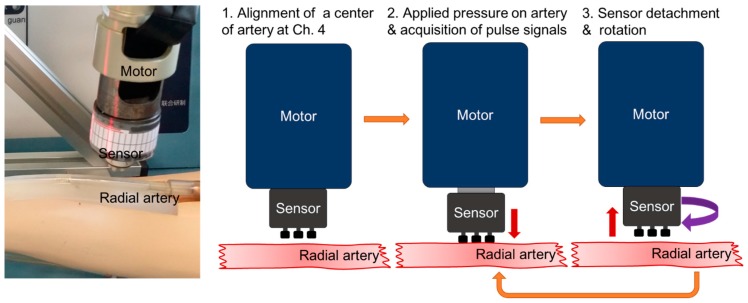
Experimental sequence for estimating blood vessel direction on the artery model in the simulator.

As shown in Figure 9b, channel four, located in the center of the PSRP, was placed at the center of the blood vessel. We assumed that channel four was placed on center of the artery when pulse signals were only received from channel four and signals from other channels were absent. The applied pressure was controlled by the motor such that the output voltage of the channel four signal reached 5 V, which is the target output voltage. The experimental sequence and method for estimating blood vessel direction in the artery model in the simulator is shown in Figure 10.

The signals from each channel were obtained for more than 20 s (sampling rate: 200 samples/s). After signal acquisition was complete, the PSRP was lifted, rotated by 5°, and then applied again. This process of the experiment was repeated until the degree of rotation reached 60° because the degree gap between channels is 60°. The obtained signals were separated into applied pressure signals and pulse waveform signals for analysis according to the PSRP sensor rotation.

## 3. Results and Discussion

### 3.1. Characteristics of PSRP Sensor

To evaluate the developed sensor, the coefficient of variance (CV) for experiments that were repeated three times was calculated, and the sensitivity per channel was obtained by increasing the applied force by seven stages. The sensitivity was calculated using the output signals of the sensors, and the repeatability was evaluated using the CV of the five sensors, as shown in Table 1. The average sensitivity of the five sensors was 0.327 (V/gf), and the average CV was 0.62%. In detail, the sensitivity corresponds to 0.016 V per 1 mmHg because a force of 10 gf is equivalent to a pressure of 203.760 mmHg. Considering the CV, the developed sensor system has a variation of 0.245 mmHg to measure 40 mmHg. Moreover, it implies that 40 mmHg, which is a typical pulse pressure, can be digitized into 2200 levels because the system resolution is 0.3 mV considering the supply voltage (±10 V) and the 16-bit A/D converter. The sensitivity variation and system resolution of the fabricated PSRPs are suitable for detecting various pulse contours for radial pulse diagnosis.

### 3.2. Experimental Result for Estimating Blood Vessel Direction

**Figure 11:**
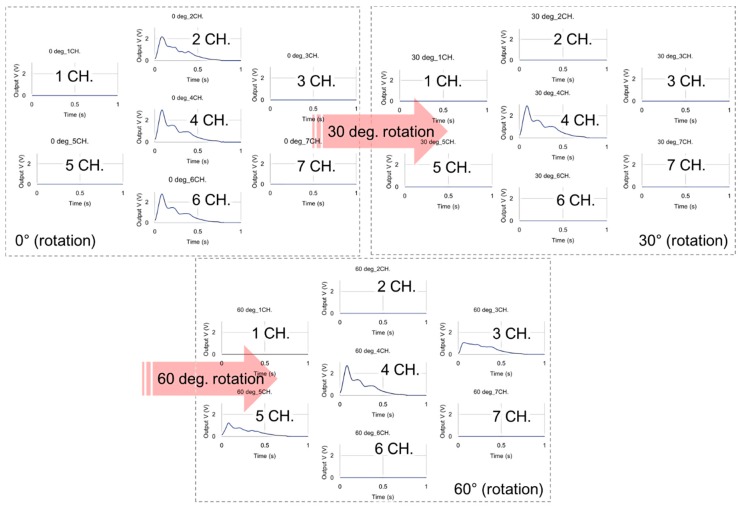
Average waveform of the pulse signal obtained from each channel according to PSRP rotation.

**Figure 12:**
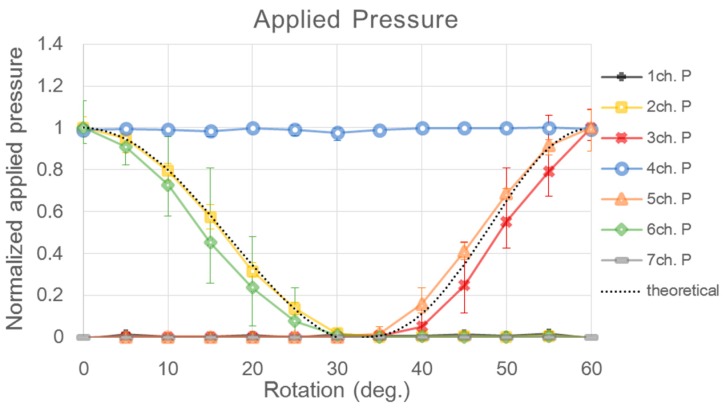
Normalized output signal for pressure value of each sensor channel with respect to sensor rotation from 0° to 60°.

The signal obtained by the sensor was divided into an applied pressure signal and a pulse waveform signal. The applied pressure signal, according to sensor rotation, was calculated by the average value of the applied pressure signals obtained for 20 s. The peak value of the pulse waveform, according to sensor rotation, was calculated from the average waveform, after deriving the average waveform from the obtained pulse waveform signals as shown in Figure 11. Theoretical values were calculated by substituting the information on the sensor location and the blood vessel geometry of the simulator into the theoretical equation in the experiment for estimating the blood vessel direction. Figure 12 is a graph of the normalized applied pressure, according to sensor rotation, which was obtained through the experiment. The black dotted line in Figure 12 shows the theoretical values of the applied pressure signals calculated using the theoretical equation. The generally positive match between the experimental values and the theoretical values shows that the equation is adequate for expressing the force according to sensor rotation.

**Figure 13:**
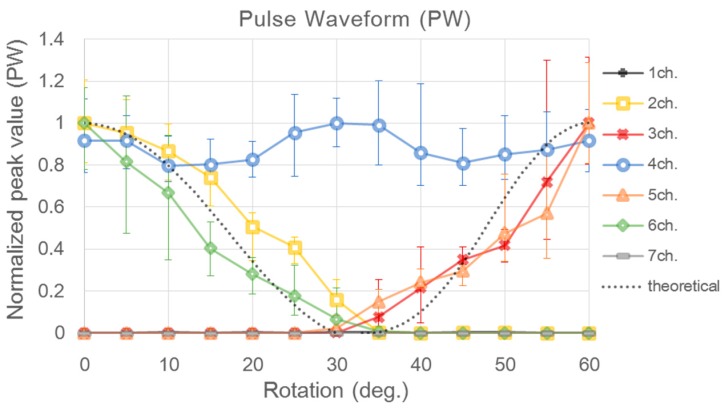
Normalized peak value of pulse wave signal for each sensor channel with respect to sensor rotation from 0° to 60°.

If the fabricated PSRP is used to measure pulse waves on the radial arteries of the body, then the blood vessel direction cannot be estimated using the applied pressure signals because the blood vessel is under the skin. Hence, the blood vessel direction needs to be estimated from the pulse waveform signal. To determine whether it is possible to estimate the blood vessel direction from the pulse waveform signal, the pulse waveform signals obtained from the experiment were compared with the theoretical value. Figure 13 depicts the peak value of the average waveform, according to sensor rotation, by deriving the average waveform from the pulse waveform signal. The black dotted line represents the values calculated from the theoretical equation. Compared with the pulse waveform signals, it has greater fluctuation and error than the applied pressure signal. The signal amplitude, according to sensor rotation, however, tends to follow the theoretical values. For instance, at 0°, where channels two, four, and six pressure the blood vessel in a straight line, the pulse waveform signal is high in all three channels. When the sensor is rotated by approximately 30°, the pulse waveform signals appear only on channel four. At 60° of rotation, the signal amplitudes are the highest in channels three, four, and five. This result shows that the pulse waveform signal of each channel fluctuates according to the sensor rotation. Based on this result, the possibility of estimating the blood vessel direction by sensor rotation was confirmed. Additionally, in the applied pressure signal graph (Figure 12), the output value of the signals from channels two and six becomes 0 at approximately 30° of sensor rotation, whereas the pressure value of channels three and five becomes 0 at approximately 35°. This is an error caused by the interval between channels two and four and between four and six, being 0.4 mm smaller than the intervals between channels three and four and four and five.

The errors in the signal are generated by the unevenness and the deformation of the simulator blood vessel by the applied pressure. The other reason is the clearance between the stick pads on the IPU and the outer case holes. The cap of the PSRP sensor does not hold uniform intervals between the stick pads when applying pressure, and the sensors apply pressure in an unwanted direction. Therefore, it is not easy to precisely estimate the blood vessel direction within 10° due to errors in the measuring signal. Due to the unevenness or deformation of the blood vessel not being within the range of control, minimization of the clearance between the stick pad and the outer case hole is required through precise assembly of the stick pad and the sensor housing case. In addition, the performance of the sensor can be improved when the contact surface between the IPU and the stick pad is flatter.

A pulse waveform that does not consider blood vessel direction can become a dominant source of error when calculating the diagnostic index using the amplitude of the pulse wave, such as AIx or pulse power [17,18]. Accurate and precise measurement of the pulse waveform is particularly important in the pulse diagnosis for Eastern medicine. The pulse sensor array must be laid 90° to the longitudinal direction of the blood vessel to accurately estimate the pulse width or length. Since the structure of the radial artery is anatomically covered by the skin layer, however, the amplitude of the pulsation needs to be compensated for when estimating the blood vessel direction. As shown in the experimental results, depending on the direction of the blood flow, an error occurs at a maximum of 10 ± 0.45 (%) from the reference point of 0° according to degree. The estimation of blood vessel direction allows the pulse width to be measured more precisely, and the developed sensor can measure the pulse width and blood vessel direction within an error of 10°. Using the fabricated sensor allows automatic determination of the direction in which the pulse sensor is laid, as it approximately infers the blood vessel direction through function approximation after measuring the pulse peak by rotating the sensor from 0 to 60°.

## 4. Conclusions and Outlook

This study developed a circular seven-channel piezoresistive sensor array using sensors assembled by the FDB method. The fabricated sensor was designed such that the pressure delivery path of each channel is independent to remove crosstalk among channels. By comparing the signal obtained from the circular-type PSRP sensor, it was possible to more precisely measure the pulse waveform by more effectively estimating the path of the radial artery. The fabricated sensor was confirmed to guarantee repeatability on sensitivity through its variance error of 0.62%, on average. The applied pressure value and peak value of the pulse waveform according to the blood vessel direction was observed using the pulse simulator to investigate the distortion effects of the blood vessel direction on the pulse waveform measurement. The results showed that it was similar to the theoretical model of pulse waveform change due to blood vessel direction and the relative difference in rotation angle (*Ɵ*) of the pulse sensor.

Applying pressure to the blood vessel in a certain direction when the radial artery is not visible is one of the essential factors for obtaining more precise pulse waveforms. The fabricated sensor allows improvement of the reliability of the pulse measurement because the PSRP sensor can be measured in the same direction of the blood vessel whenever measuring the pulse wave. Moreover, it is expected to improve the diagnostic precision of pulse indices that use the change in pulse amplitude, such as AIx, pulse width, and pulse power, which have high clinical usage in Eastern and Western medicine.

Studies on computational methods that estimate the blood vessel direction and compensate for the pulse wave error using the approximation model of the blood vessel direction that we developed are required as follow-up investigations. Moreover, additional analysis on the factors that cause measurement errors (e.g., structural modification of the radial artery according to the applied pressure, mechanical errors in the production process of the pulse sensor, deformation noise during applied pressure to the radial artery due to soft tissue characteristics of the blood vessel and the skin layer) need to be conducted. Finally, the possibility of practical utilization of the fabricated sensor needs to be studied by actually applying the sensor in clinics.

## Figures and Tables

**Table 1:** Sensor sensitivity.

Sensor		CH1	CH2	CH3	CH4	CH5	CH6	CH7
Sensor #1	Sensitivity (V/gf)	0.32294	0.37252	0.36359	0.35303	0.32533	0.31089	0.32765
	CV (%)	0.57954	0.03916	0.73674	0.48756	0.05013	3.05291	0.88291
Sensor #2	Sensitivity (V/gf)	0.32178	0.32949	0.33023	0.35977	0.35673	0.3545	0.34215
	CV (%)	2.38609	0.22454	0.40759	0.04822	0.08609	0.06177	0.13737
Sensor #3	Sensitivity (V/gf)	0.32415	0.35095	0.32455	0.3523	0.36408	0.32426	0.348
	CV (%)	0.22802	0.177	0.05883	0.07506	0.45001	0.21664	0.24718
Sensor #4	Sensitivity (V/gf)	0.33615	0.32551	0.25834	0.31423	0.30729	0.3162	0.3162
	CV (%)	0.22001	0.05361	0.31644	0.45993	0.43231	1.08301	0.15042
Sensor #5	Sensitivity (V/gf)	0.33256	0.30244	0.28766	0.33387	0.29701	0.27744	0.29768
	CV (%)	2.69237	0.43782	1.07538	2.37248	0.82258	0.07627	0.13191

R^2^ mean and standard deviation are 0.999168 and 0.000931, respectively.
